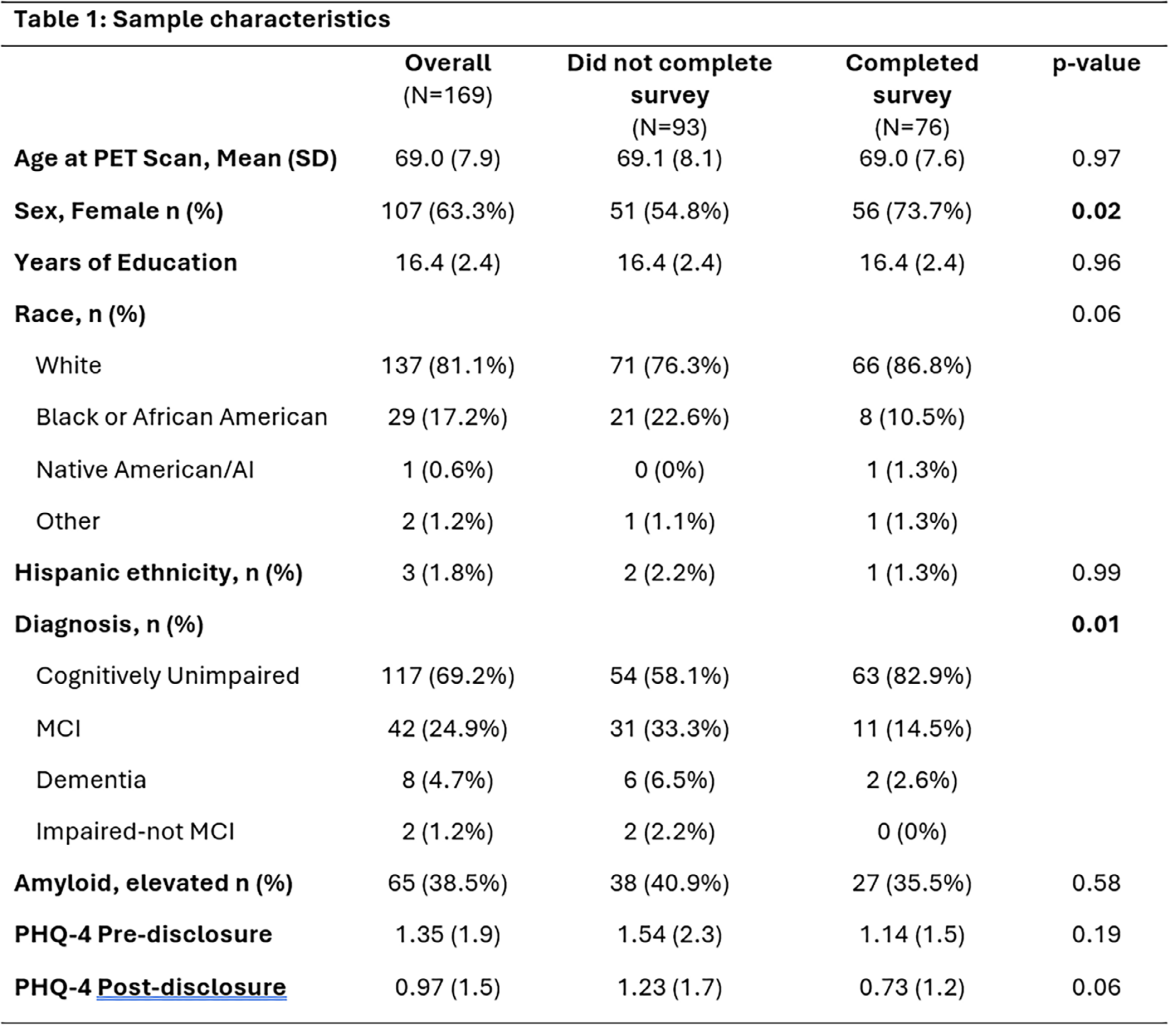# Satisfaction and utility in learning research amyloid PET results across the clinical spectrum

**DOI:** 10.1002/alz70858_105168

**Published:** 2025-12-26

**Authors:** Lindsay R Clark, Nathaniel A. Chin, Sara Frost Alberson, Fred B. Ketchum, Kristin E Basche, Hannah Rosario, Amanda Peterson, Isa D Hayde, Melissa Bahr, Madilynn Wintlend, Olivia R Mandel, Marcella Eveler, Cara R Omernik, Sterling C Johnson

**Affiliations:** ^1^ University of Wisconsin‐Madison School of Medicine and Public Health, Madison, WI, USA

## Abstract

**Background:**

Communicating Alzheimer's disease (AD) biomarker results with adults enrolled in observational cohorts is of interest to participants and researchers. The objective of this project is to evaluate participant experience and satisfaction with learning amyloid PET results.

**Method:**

Amyloid PET results were provided to 169 late middle‐aged and older adults enrolled in observational cohorts (mean age=69 [SD=7.9]; 63% women; 19% from under‐represented groups [URGs]; 31% cognitively impaired [CI]) (Table 1). Optional surveys assessing clinical and personal utility, result comprehension, clinician competence, and satisfaction were completed approximately 4‐6 months after result disclosure. Summary statistics were computed for each survey item. T‐tests or Mann‐Whitney U tests were conducted to compare responses by amyloid result (elevated, non‐elevated) and cognitive status (unimpaired, impaired).

**Result:**

Seventy‐six participants completed surveys (mean age=69 [SD=7.6]; 74% women; 13% URG; 17% CI). Men or those with CI were less likely to complete surveys. Of survey respondents, 92‐100% endorsed items regarding usefulness of educational materials and clinician competence, and 93% understood the meaning of their PET result. Participants endorsed greater willingness to complete the PET scan because they could learn the result (59%) and most felt it was the right decision (95%), useful (82%), and shared results with others (87%). Respondents endorsed lifestyle behavior change (57%), advanced planning (24%), or making an appointment with a healthcare provider (24%) after learning results, and 41% endorsed interest in enrolling in AD clinical trials. Participants with CI, compared to unimpaired adults, were less likely to understand educational materials (*p* = .01) and the meaning of their PET result (*p* = .05) and more likely to make a healthcare appointment (*p* = .001) or changes to advanced planning (*p* = .01). CI participants, as well as participants with elevated PET results, were more interested in AD clinical trials (*p* < .01).

**Conclusion:**

Adults with and without CI were satisfied and found learning AD research biomarker results useful. Improvements to materials or processes are needed to ensure comprehension of results for individuals with CI. Barriers to survey completion by gender or cognitive status need to be identified and addressed to ensure representative feedback. More research is needed to assess actual post‐disclosure behavioral changes and barriers.